# Reassessment of the DTI-ALPS index in focal epilepsy

**DOI:** 10.1186/s41747-026-00799-8

**Published:** 2026-10-23

**Authors:** Stefanie Chambers, Leo Hofer, Mario Tranfa, Patric Kienast, Julia Shawarba, Radheshyam Stepponat, Judith Jud, Susanne Aull-Watschinger, Ekaterina Pataraia, Florian Ph.S Fischmeister, Frederik Barkhof, Gilbert Hangel, Karl Rössler, Giuseppe Pontillo, Gregor Kasprian, Lukas Haider

**Affiliations:** 1 Department of Biomedical Imaging and Image-Guided Therapy, Medical University of Vienna, Vienna, Austria; 2 Department of Neurosurgery, Medical University of Vienna, Vienna, Austria; 3 Christian Doppler Laboratory for MR Imaging Biomarkers, Medical University of Vienna, Vienna, Austria; 4 Comprehensive Center for Clinical Neurosciences & Mental Health, Medical University of Vienna, Vienna, Austria; 5 Department of Advanced Biomedical Sciences, University of Naples Federico II, Naples, Italy; 6 Department of Radiology and Nuclear Medicine, Amsterdam UMC, Vrije Universiteit, Amsterdam, Netherlands; 7 Department of Neurology, Medical University of Vienna, Vienna, Austria; 8 UCL Queen Square Institute of Neurology, University College London, London, UK; 9 UCL Hawkes Institute, Department of Medical Physics and Biomedical Engineering, University College London, London, UK; 10 Department of Neuroradiology, Karolinska University Hospital, Stockholm, Sweden

**Keywords:** Diffusion tensor imaging, Epilepsy (partial), Glymphatic system, Malformations of cortical development, Magnetic resonance imaging

## Abstract

**Objective:**

Recent evidence highlights the limited specificity of diffusion tensor imaging (DTI) along perivascular spaces (ALPS) as a noninvasive glymphatic biomarker because microstructural tract degeneration and crossing-fiber geometry confound it. We aimed to reassess the interpretation of the DTI-ALPS index in people with focal epilepsy.

**Materials and methods:**

Seventy-nine people with drug-resistant focal epilepsy (PWFE) underwent comprehensive presurgical work-up and 3-T magnetic resonance imaging (MRI). Etiologies included MRI-negative epilepsy, malformations of cortical development, temporal lobe epilepsy, and low-grade epilepsy-associated tumors. DTI preprocessing was conducted using QSIPrep with automated region-of-interest (ROI) placement. Linear regression models and Wilcoxon signed-rank tests were used to assess group differences and associations with clinical variables. Twenty-five age- and sex-matched healthy controls were included for comparison.

**Results:**

Seventy-nine patients (28.6 years ± 11.3 [mean ± standard deviation]; 47 men, 32 women) and 25 healthy controls (mean age, 26.7 years ± 4.2; 11 men, 14 women) were evaluated. DTI-ALPS findings were more abnormal in lesions in proximity to the ROI (*p* = 0.014), implicating local white matter disruption as a modifier, which was further corroborated by reduced postoperative values. No associations were identified with disease duration, sex, or normalized gray-or white matter volumes.

**Conclusion:**

Our results demonstrate a distance-dependent correlation of DTI-ALPS values to the seizure onset zone in focal epilepsy, whilst no significant associations to disease duration or normalized brain volumes were found. Together, these findings suggest that local disruption of fiber tracts may act as important modifiers of this measure and warrant caution in the interpretation of the DTI-ALPS index.

**Key Points:**

***Question*** Microstructural changes may significantly confound the interpretation of the DTI-ALPS index as a surrogate parameter for glymphatic system dysfunction in epilepsy.

***Findings*** No significant differences in the DTI-ALPS index were observed comparing PWFE with healthy controls; however, lesion proximity and post-operative status significantly alter the metric.

***Relevance statement*** Local white-matter disruption and microstructural architecture may significantly alter the DTI-ALPS index; therefore, caution is warranted when interpreting the index as a glymphatic biomarker in focal epilepsy.

**Graphical Abstract:**

**Figure:**
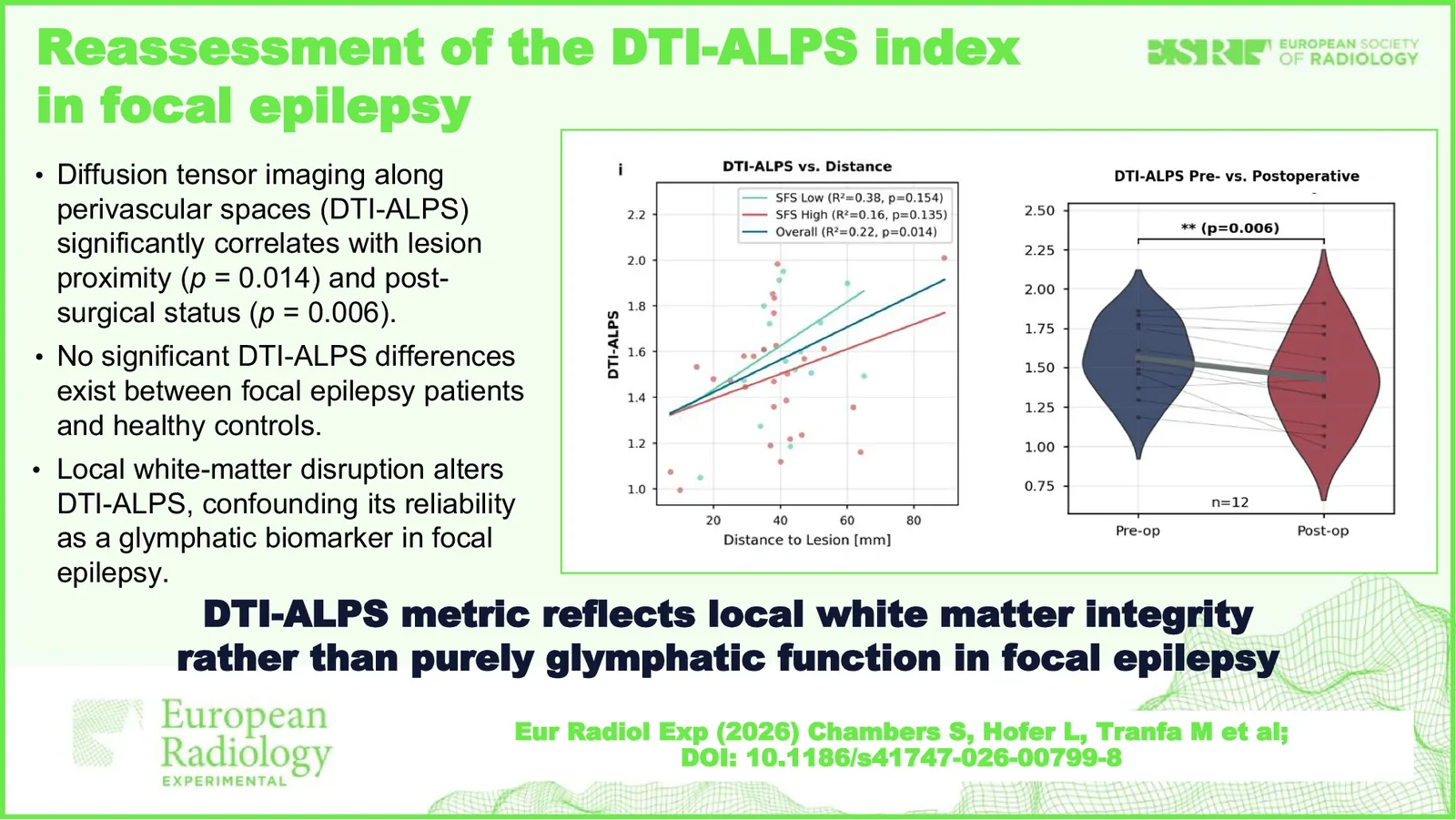

## Background

Epilepsy is a chronic illness caused by a wide array of underlying pathologies giving rise to neuronal hyperexcitability and synchronicity, culminating in its unifying clinical symptom, seizures [1]. Exposure to such synchronized hyperexcitability has multiple effects on neurons, ranging from glutamate excitotoxicity [2] to increased neurotransmitter exchange in the synaptic cleft and blood-brain barrier leakage [3]. It has been proposed that this leads to altered cerebral interstitial fluid flux, allowing for protein accumulation [2]. The glymphatic system constitutes a key mechanism in the clearance of metabolic waste products, amongst many of its proposed functions in maintaining brain health. However, glymphatic system dysfunction it has not only been implicated as a consequence of epilepsy, but also as a cause of hyperexcitability [4, 5].

Evidence implies the conversion of multiple pathways, such as aquaporin-4 (AQP4) mislocalization [6, 7], inflammation due to accumulation of waste products, catalyzing neurodegeneration and mTOR activation [8], and extracellular ion alterations, facilitating epileptogenesis.

Noninvasive measures for the glymphatic system have remained limited [9], and the diffusion tensor imaging analysis along the perivascular space (DTI-ALPS) index [10, 11], which leverages the unique orientation of medullary vessels, projection and association fibers at a specific level adjacent to the lateral ventricles, had initially been propagated as a potential surrogate parameter. Multiple groups have demonstrated decreased DTI-ALPS index values across neurodegenerative disorders such as Alzheimer [10] and Parkinson [12, 13] disease or in epilepsy [14–19], concluding that associated neuronal changes may potentially be related to glymphatic system dysfunction. However, a growing body of evidence suggests that the DTI-ALPS metric is not specific to glymphatic system dysfunction and is in part affected by degenerative changes of microstructural tissue integrity related to natural aging, even in areas perpendicular to medullary veins [20]. Recent studies suggested that DTI-ALPS was a poor surrogate for glymphatic function, showing the index primarily reflected vascular pathology. Experimental evidence further revealed that crossing fibers systematically inflate the DTI-ALPS index through mathematical bias, undermining the method’s validity as a glymphatic biomarker [21, 22]. This has led the authors of the original manuscript to revisit the definition of the DTI-ALPS metric, underpinning that the interpretation of the DTI-ALPS index as a parameter for glymphatic system function should be used with caution [11].

Given these emerging concerns about the validity and specificity of DTI-ALPS as a glymphatic biomarker, there is a pressing need to reassess its clinical utility in epilepsy.

The objective of this study is to systematically evaluate the DTI-ALPS index in a large cohort of people with focal epilepsy (PWFE), including both magnetic resonance imaging (MRI)-negative cases and patients with diverse structural pathologies. We assessed clinical data and potential confounding factors, such as lesion proximity to the DTI-ALPS region of interest (ROI) and global brain volume, to advance understanding of how these factors influence the DTI-ALPS index.

## Materials and methods

### Ethics approval

Institutional Review Board approval was obtained for this retrospective study (EK Nr. 1604/2018).

### Patient selection criteria and clinical work-up

Following institutional review board approval, this retrospective analysis was conducted on drug-resistant PWFE who had been evaluated as surgical candidates from September 2019 to March 2023 at the Medical University of Vienna following informed written consent.

The comprehensive clinical work-up included prolonged video-electroencephalogram monitoring, state-of-the-art 3-T MRI protocol for epilepsy in accordance with the HARNESS protocol [23], functional MRI (fMRI), and neuropsychological testing. Patients with concomitant neurological diseases, a history of chemotherapy, or prior brain surgery were excluded from the analysis. Following retrospective chart review, patients were grouped into etiological groups, namely lesional (*i.e*., radiologically suspected or histopathologically verified lesion) and nonlesional (*i.e*., MRI-negative) epilepsy. Lesional pathologies included malformations of cortical development, low-grade epilepsy associated neuroepithelial tumors (LEAT), hippocampal sclerosis, and encephaloceles, which were subsumed as lesional temporal lobe epilepsy (lesional TLE). The suspected seizure onset zone (SOZ) as defined in the comprehensive clinical workup was extended to a lobar level (*i.e*., temporal, frontal) in our analyses. Lateralization (right, left) refers to the affected hemisphere.

To assess epilepsy-related disease-specific effects on the DTI-ALPS index, we further included disease duration (defined as duration from initial diagnosis to time point of measurement) and seizure frequency using the seizure frequency score (SFS) [24] at the time point of MRI measurement, as clinical variables. We consolidated scores ≥ 7 (*i.e*., at least monthly occurring seizures) into the high seizure frequency group, and < 7 (less than monthly occurring seizures) into the low seizure frequency group. We intentionally refrained from calculating cumulative seizure burden due to the difficulty of reliably quantifying this measure. Postoperative outcome was assessed according to the ILAE outcome scale [25]. The control group consisted of all available patients in our institution with matching MRI protocols acquired on the same scanner, which limited the number of eligible controls to 25.

### Data acquisition

All scans were performed on the same 3-T scanner (Ingenia Elition, Philips, Amsterdam, The Netherlands). DTI was acquired with 64 different diffusion directions (echo time = 89 ms, repetition time = 3,175.7 ms, flip angle = 90°, slice thickness = 2 mm, field of view = 224 × 224 mm^2^, and *b*-value = 1,000 s/mm^2^). Volumetric analysis was performed on T1-weighted imaging (echo time = 3.7 ms, repetition time 8.2 ms, flip angle = 8°, slice thickness = 1 mm).

### Segmentation and ALPS calculation

We used QSIPrep v0.19.0 [26] for diffusion MRI preprocessing, using default parameters. Then, following a previously validated workflow, the fractional anisotropy (FA) map and *x*-, *y*-, and *z*-axis diffusivity maps were generated using FSL’s dtifit [27], the FA map of each subject was co-registered to the JHU-ICBM-FA template via FSL’s flirt, and the transformation matrix was applied to all the diffusivity maps. Regions of interest (ROIs) were automatically defined in the template space as 5-mm spheres within the bilateral projection and association fibers of the superior corona radiata and the superior longitudinal fasciculus (identified based on the JHU-ICBM-DTI-81-white-matter labeled Atlas) at the level of the lateral ventricle body, and applied to all subjects’ diffusivity maps [28]. The DTI-ALPS index was calculated according to the formula as previously proposed [10]. Segmentation of three-dimensional T1-weighted sequences was performed using SamSeg [29]. Total gray- and white matter volume was normalized to (*i.e*., divided by) total intracranial volume to obtain the proportional volumes of gray and white matter relative to total cranial volume. Distance measurements between the DTI-ALPS sample region (peri-ventricular region on the lesional side) and the closest border of MRI-visible lesions were performed manually by S.C. and L.Ho. in consensus.

### Statistical analysis

**Fig. 1:**
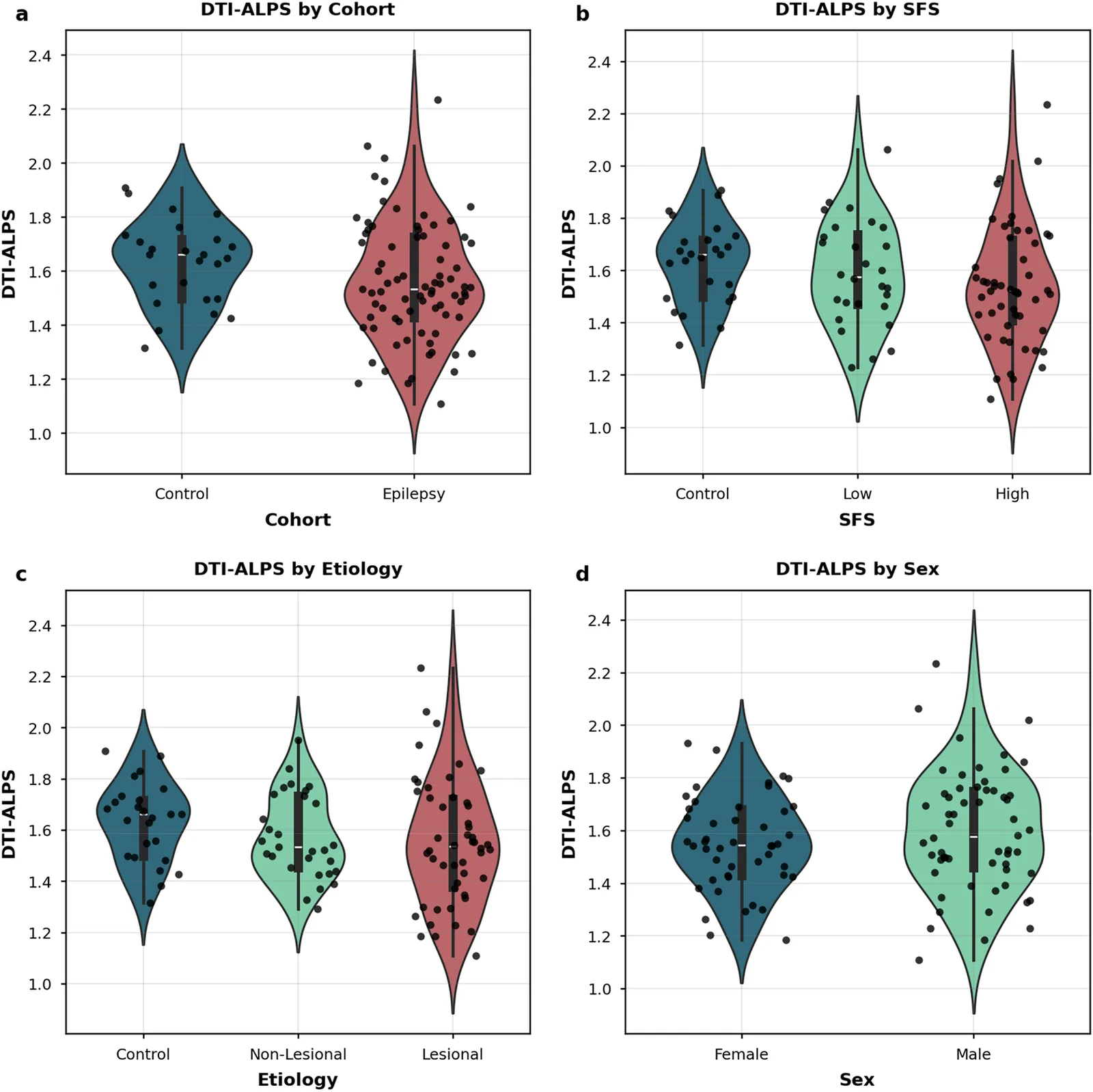
DTI-ALPS score across clinical variables. Violin plots showing the distribution of DTI-ALPS values across clinical subgroups. **a** Comparison between PWFE and healthy controls. **b** Stratification by SFS (control, low, and high). **c** Stratification by etiology (control, non-lesional, and lesional). **d** Comparison by sex (female and male). Box plots within each violin display the median (central line), interquartile range (box), and whiskers extending to 1.5 times the interquartile range with individual data points overlaid. Group-level statistical inference was performed using a single multivariable linear regression model with SFS group, age at MRI, and sex as pre-specified predictors. DTI-ALPS, Diffusion tensor imaging along perivascular spaces; MRI, Magnetic resonance imaging; SFS, Seizure frequency score

**Fig. 2:**
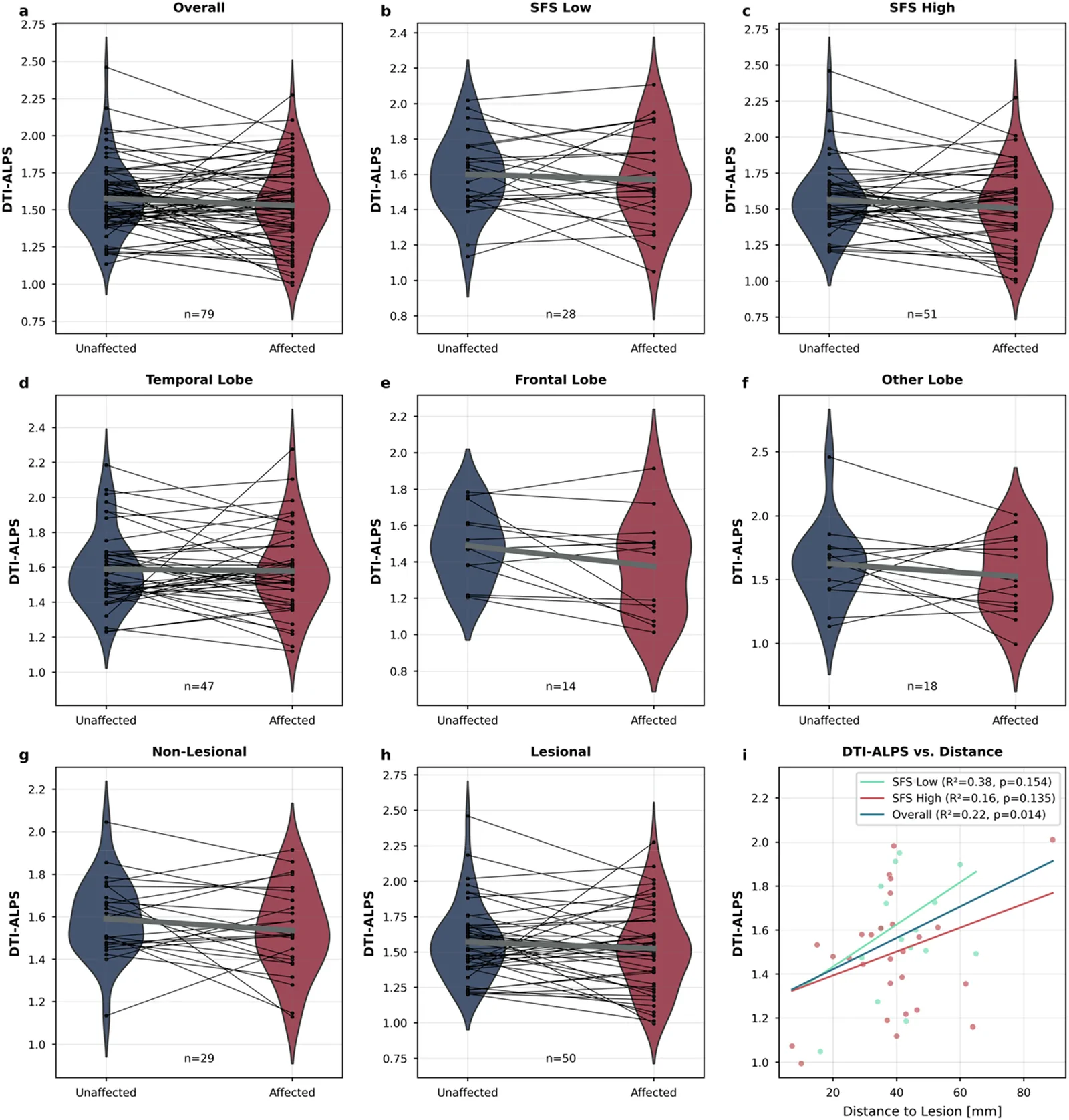
Hemispheric DTI-ALPS comparison across brain regions and epilepsy etiologies. Comparative analysis of DTI-ALPS values between affected and unaffected hemispheres. **a** Overall comparison showing no significant changes in DTI-ALPS values comparing affected *versus* unaffected hemispheres (β = -0.05, *p* = 0.368). **b**, **c** Individual analysis of hemispheric differences stratified by SFS did not show significant differences for high (β = -0.03, *p* = 0.594) and low (β = -0.06, *p* = 0.368) SFS. **d**–**f** Regional analysis by brain lobe revealed no significant hemispheric differences in the frontal (β = -0.11, *p* = 0.368), temporal (β = -0.10, *p* = 0.771) or “other” lobes (β = -0.01, *p* = 0.387). **g**, **h** Etiology-based hemispheric comparisons showed no significant differences for lesional (β = -0.04, *p* = 0.387) or non-lesional epilepsy (β = -0.06, *p* = 0.368). **i** The DTI-ALPS score was significantly associated with distance to the lesion in the overall epilepsy cohort (β = 0.01, *p* = 0.014); individual analysis of high- or low-seizure frequency subgroups did not show significant associations with distance. Violin plots display distributions with median (gray line) and individual paired values (black lines). β, Coefficient; DTI-ALPS, Diffusion tensor imaging along perivascular spaces; *n,* Sample size; SFS, Seizure frequency score

All statistical analyses were performed using Python 3.14 (scipy v1.15, statsmodel v0.14). Normality of data distribution was assessed using the Shapiro-Wilk test, supporting the use of parametric methods (linear regression and linear mixed models). For the paired preoperative/postoperative subgroup (*n* = 12), where small sample size limits the reliability of normality testing, the Wilcoxon signed-rank test was used. A multivariable linear regression model with DTI-ALPS as the dependent variable was fitted and backward elimination applied with pre-specified predictors (SFS group, age at MRI and sex). Stratified analyses of subgroups defined only within the epilepsy cohort (suspected SOZ lobe, etiology subtype) and paired hemispheric comparisons were performed using linear mixed models with patient as a random effect. Multiple comparisons were addressed using the Benjamini-Hochberg false discovery rate (FDR) procedure, applied separately within each family of related tests: across the four coefficients of the primary multivariable model (Fig. 1), and across the stratified hemispheric and subgroup comparisons (Fig. 2). Both raw and FDR-adjusted exact *p*-values are reported.

## Results

A total of 104 participants were included in the final analysis, comprising 79 PWFE (Table 1) and 25 healthy controls. PWFE had an age of 28.6 ± 11.3 years (mean ± standard deviation [SD]) and included 47 males (59.5%) and 32 females (40.5%). The control group had an age of 26.7 ± 4.2 years and included 11 males (44%) and 14 females (56%). MRI-negative cases were the most common (*n* = 27, 34.2%), followed by malformations of cortical development (*n* = 20, 25.3%), lesional TLE (*n* = 15, 19%), and LEAT (*n* = 14, 17.7%), with other etiologies (infarction, vascular pathologies) comprising 3.8% (*n* = 3). The temporal lobe was the most frequent SOZ (*n* = 47, 59.5%) followed by the frontal lobe (*n* = 14, 17.7%), while the remaining (parietal, occipital, or unclear) lobes were subsumed into “other” (*n* = 18, 22.8%). 51 PWFE (64.6%) were classified as having high seizure frequencies, with the remaining 28 (35.4%) showing low seizure frequencies. The mean duration of epilepsy was 12.3 ± 11.2 years (Table 1).

**Table 1:** Cohort summary (*n* = 79)

Age (years)	28.6 ± 11.3
Sex	
Male	47 (59.5%)
Female	32 (40.5%)
Etiology	
MRI negative	27 (34.2%)
Malformation of cortical development	20 (25.3%)
TLE	15 (19.0%)
LEATs	14 (17.7%)
Other	3 (3.8%)
Suspected SOZ	
Temporal	47 (59.5%)
Frontal	14 (17.7%)
Other	18 (22.8%)
Seizure frequency	
High	51 (64.6%)
Low	28 (35.4%)
Controls	0 (0.0%)
Duration of epilepsy (years)	12.3 ± 11.2

Demographic and clinical characteristics of PWFE included in the DTI-ALPS analysis. Data are presented as mean ± SD for continuous variables and absolute number (percentage) for categorical variables

*DTI-ALPS* Diffusion tensor imaging along perivascular spaces, *LEAT* Low-grade epilepsy-associated neuroepithelial tumors, *MRI* Magnetic resonance imaging

Multivariable linear regression analysis with the pre-specified predictors seizure frequency, age at MRI, and sex did not reach statistical significance overall (adjusted *R*² = 0.030, *p* = 0.133).

Among individual predictors, only the SFS High group showed a significant association, with lower DTI-ALPS values compared to controls (β = -0.102, 95% CI: -0.20 to -0.002, *p* = 0.045, FDR-adjusted *p* = 0.18). The SFS Low group did not differ significantly from controls (β = -0.055, 95% CI: -0.17 to 0.06, *p* = 0.337, FDR-adjusted *p* = 0.34), nor did age at MRI (β = 0.003, *p* = 0.143, FDR-adjusted *p* = 0.25) or sex (β = 0.054, *p* = 0.190, FDR-adjusted *p* = 0.25).

Backward elimination was performed on the same set of predictors. In the final model, only SFS was retained (*p* = 0.081), converging with the primary analysis in identifying SFS as the principal patient-level predictor. Descriptive subgroup distributions are summarized in Fig. 1 and Table 2.

**Table 2:** Summary statistics of DTI-ALPS score across clinical variables

Panel	Group	*n*	Mean ± SD
DTI-ALPS by cohort	Control	25	1.631 ± 0.155
	Epilepsy	79	1.560 ± 0.219
DTI-ALPS by SFS	Control	25	1.631 ± 0.155
	Low	28	1.591 ± 0.201
	High	51	1.543 ± 0.228
DTI-ALPS by etiology	Control	25	1.631 ± 0.155
	Nonlesional	29	1.574 ± 0.166
	Lesional	50	1.552 ± 0.246
DTI-ALPS by sex	Female	46	1.552 ± 0.176
	Male	58	1.598 ± 0.228

Group-wise count (*n*), mean ± SD of DTI-ALPS scores across four clinical stratifications

*DTI-ALPS* Diffusion tensor imaging along perivascular spaces, *SFS* Seizure frequency score

Next, we analyzed individual hemispheric DTI-ALPS scores. Comparing affected and unaffected hemispheres revealed no significant difference, with slightly lower mean DTI-ALPS values in the affected hemisphere (mean = 1.529, SD = 0.272), compared to 1.577 (SD = 0.239) on the contralateral side (β = -0.048, *p* = 0.368; Fig. 2a and Supplementary Table S1). Lateralization of the suspected SOZ did not significantly affect the measured DTI-ALPS score (β = 0.016, *p* = 0.760).

When stratified by seizure frequency, neither the high SFS group (affected mean ± SD = 1.506 ± 0.28 *versus* unaffected = 1.564 ± 0.252) nor the low SFS group (affected mean = 1.57 ± 0.258 *versus* unaffected = 1.6 ± 0.217) demonstrated a significant hemispheric effect (β = -0.03, *p* = 0.594 and β = -0.058, *p* = 0.368, respectively; Fig. 2b, c and Supplementary Table S1).

Regional analysis by brain lobe revealed that only PWFE with a suspected seizure onset in the frontal lobe group (affected mean ± SD = 1.375 ± 0.275 *versus* unaffected = 1.49 ± 0.199) exhibited a significant hemispheric difference (β = -0.114, *p* = 0.046; Fig. 2e), but this finding did not survive correction for multiple comparisons (FDR-adjusted *p* = 0.368). SOZ in temporal (affected mean ± SD = 1.579 ± 0.244 *versus* unaffected = 1.59 ± 0.219), (β = -0.011, *p* = 0.771) and “other” lobes (affected mean ± SD = 1.527 ± 0.312 *versus* unaffected = 1.625 ± 0.317), (β = -0.099, *p* = 0.269) showed non-significant trends in the same direction (Fig. 2d–f and Supplementary Table S1).

Etiology-based analyses showed that neither lesional epilepsy (affected mean ± SD = 1.526 ± 0.302 *versus* unaffected = 1.57 ± 0.265) nor non-lesional epilepsy (affected mean ± SD = 1.535 ± 0.208 *versus* unaffected = 1.591 ± 0.183) was associated with significant differences of DTI-ALPS values across hemispheres (β = -0.044, *p* = 0.387 and β = -0.055, *p* = 0.368, respectively; Fig. 2g, h and Supplementary Table S1).

Finally, to further assess a potential distance-dependent effect, we correlated the distance [mm] of the closest lesional border to the DTI-ALPS ROI on the affected side in a subgroup of MRI-visible lesions which showed an overall significant positive correlation (β = 0.007, *p* = 0.014) while individual analysis of the high- (β = 0.005, *p* = 0.667) and low- (β = 0.010, *p* = 0.154) SFS group yielded non-significant correlations but following this trend (Fig. 2i and Supplementary Table S1).

**Fig. 3:**
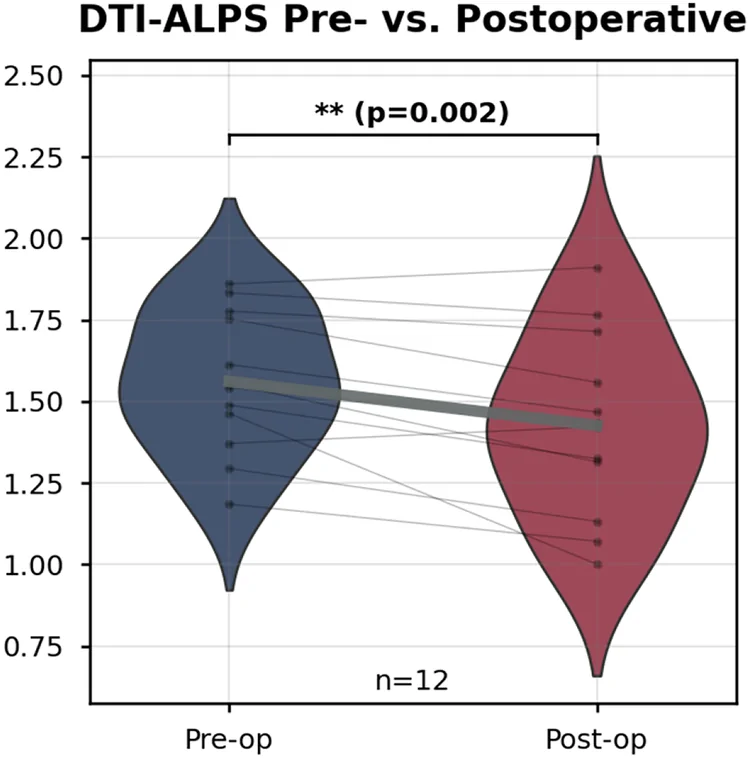
Longitudinal DTI-ALPS Score pre- and postoperatively. Violin plots of DTI-ALPS values for the subset of patients with paired pre- and postoperative scans (*n* = 12). Thin lines represent individual subject trajectories, while the thick gray line indicates the mean change across the cohort. Statistical analysis using the Wilcoxon signed-rank test revealed a significant decrease in DTI-ALPS scores following surgery (*p* = 0.002). DTI-ALPS, Diffusion tensor imaging along perivascular spaces

To investigate the longitudinal impact of surgical intervention on the DTI-ALPS score, we analyzed a subset of patients (*n* = 12) with available paired pre- and postoperative MRI data. A Wilcoxon signed-rank test revealed a significant reduction in DTI-ALPS scores following surgery (*p* = 0.002, Fig. 3).

To assess correlations of brain volume and DTI-ALPS measurements, linear regression analyses between the DTI-ALPS score normalized to total white matter and gray matter volumes, adjusting for age and sex, were performed, but did not show significant associations (Supplementary Fig. S1).

## Discussion

In this study, we provided a systematic evaluation of the DTI-ALPS index in a large cohort with focal epilepsy. While our results suggest a tendency towards lower DTI-ALPS index values in the affected hemispheres, most evident in PWFE with high seizure frequencies, this finding did not survive correction for multiple testing. We were further unable to demonstrate significant group differences in the DTI-ALPS index between healthy controls and PWFE. Conversely, lesion proximity to the DTI-ALPS ROI showed significant correlation in our cohort, implying potential local fiber-tract-associated changes as modifiers of the metric. This was further corroborated by reduced post-operative DTI-ALPS values. Together, these findings suggest that both lesion-related microstructural changes and post-surgical structural changes are associated with reduced DTI-ALPS values, highlighting its sensitivity to local tissue architecture.

These results highlight the necessity to exert caution in the interpretation of the DTI-ALPS index in the context of epilepsy. Though the tendency for decreased DTI-ALPS indices with higher seizure frequencies and frontal lobe involvement aligns with prior reports linking greater seizure burden to frontal lobe onset [30, 31], our findings suggest that focal structural abnormalities may disrupt white matter integrity in ways that violate the underlying assumptions of the DTI-ALPS metric. Previous research has demonstrated that lesional epilepsies, particularly focal cortical dysplasia [32], showed lower values compared to non-lesional cases, and supporting this, we found that proximity of the lesion to the DTI-ALPS ROI was positively correlated with the DTI-ALPS index, suggesting that this may act as an important modifier of the measure. Further supporting the notion of only minor influence of symptomatic epilepsy relative to the inherent predominant impact of the underlying structural pathology on the DTI-ALPS index, is the fact that we were unable to identify significant correlations between the index and disease-specific clinical variables such as duration, in line with previous research [14–16, 33].

We were unable to demonstrate significant group differences in the DTI-ALPS index between PWFE and healthy controls. Though this stands in contrast to several prior reports [15, 16, 33], our findings are not without precedent. The to-date largest study of the DTI-ALPS index in a normative cohort [14] was similarly unable to detect group-level differences between healthy controls and PWE, instead demonstrating that age was the main predictor of the index in their analysis. The absence of an age correlation in our study is likely explained by the comparatively young mean age of our cohort, which was below 40 years, the age at which DTI-ALPS indices are thought to peak [34].

In this study, it remains difficult to determine whether changes in the DTI-ALPS measure specifically reflect glymphatic system dysfunction, as complementary non-invasive techniques such as Intravoxel Incoherent Motion (IVIM) or intrathecal gadolinium applications [35], were not available. Our observation, however, supports the possibility that microstructural degeneration associated with ageing [20] or local disruptions due to pathologies, rather than glymphatic dysfunction per se, accounts for at least part of the observed variance.

A limitation inherent to epilepsy research is the difficulty in objectively quantifying disease-specific variables such as seizure burden. This is further exacerbated by seizure amnesia, making underestimation likely and limiting the reliability of this parameter. We therefore quantified this measure using the SFS as a pragmatic compromise, though this approach remains subject to bias. Similarly, potential confounding effects of drug load merit consideration: prior studies have reported lower DTI-ALPS values in drug-resistant [32] compared to drug-responsive epilepsy, raising the possibility that medication exposure could obscure or amplify disease-related associations.

Our study is further limited by its cross-sectional nature, preventing assessment of longitudinal trajectories of DTI-ALPS values except for a small subgroup where postoperative MR imaging was available. Additionally, an inherent problem in all DTI-ALPS studies is the subjective selection of voxels for the placement of the DTI-ALPS ROI, restricting the comparability across studies. Furthermore, the selected ROI for the DTI-ALPS index is agnostic to the disease focus, and our results suggest that the metric is at least partially influenced by distance to ROI, warranting caution in the interpretation in focal neurological diseases.

Finally, given the high interdependence of coexisting neurodegenerative changes and disease-specific pathomechanisms, reliably disentangling these effects remains a persistent challenge inherent to this line ofresearch.

In summary, our data indicate a significant correlation of lesion proximity and postoperative status to the DTI-ALPS index, whilst showing no correlation to disease-specific variables. Together, these findings suggest that local disruption of fiber tracts may act as important modifiers of this measure and warrant caution in the interpretation of the DTI-ALPS index in the context of focal epilepsy.

## Supplementary information

**Additional file 1: Suppl. Table 1** Summary Statistics of DTI-ALPS Scores in Affected and Unaffected Hemispheres. **Suppl. Fig. 1** Association of DTI-ALPS Score with White- and Gray Matter Volume. Relationship between ALPS values and total white- and gray matter volume normalized to total intracranial volume was assessed. (**a**) No significant associations were observed between DTI-ALPS and normalized TWM in the high and low SFS groups (control: *p* = 0.601, R² = 0.11; SFS-low: *p* = 0.653, R² = 0.06; SFS-high: *p* = 0.727, R² = 0.01). (**b**) There were no significant associations between DTI-ALPS and normalized TGM in any group (control: *p* = 0.547, R² = 0.11; SFS-low: *p* = 0.724, R² = 0.05; SFS-high: *p* = 0.395, R² = 0.04). Scatterplots display linear regression fits for each group: control (blue), SFS-low (green), and SFS-high (red). Lines represent the predicted values from ordinary least squares regression, adjusted for age and sex. **Suppl. Fig. 2** Association of DTI-ALPS Score with ILAE Outcome. No significant association between the DTI-ALPS score and ILAE outcome score dichotomized into 1 (*i.e.* no seizures after intervention) and ≥2 was found. Box plots display median (central line), interquartile range (box), and whiskers extending to 1.5 times the interquartile range.

## Data Availability

Data supporting the findings of this study are available from the corresponding author upon reasonable request.

## Abbreviations

DTI: Diffusion tensor imaging
DTI-ALPS: DTI along perivascular spaces
FA: Fractional anisotropy
FDR: False discovery rate
LEAT: Low-grade neuroepithelial epilepsy-associated tumor
MRI: Magnetic resonance imaging
PWFE: People with focal epilepsy
ROI: Region of interest
SD: Standard deviation
SFS: Seizure frequency score
SOZ: Seizure onset zone
TLE: Temporal lobe epilepsy
