# Supplementary material for: Reassessment of the DTI-ALPS index in focal epilepsy
Source: Eur Radiol Exp. 2026 Oct 23;10:162. doi: 10.1186/s41747-026-00799-8 (PMC13694682; doi:10.1186/s41747-026-00799-8)
Supplement: Supplementary file 1 [file 41747_2026_799_MOESM1_ESM.docx]

| **Group** | **Hemisphere** | **n** | **Mean** | **standard deviation** | **Mean ±  standard deviation** |
| --- | --- | --- | --- | --- | --- |
| Overall | Unaffected | 79 | 1.577 | 0.239 | 1.577 ± 0.239 |
|  | Affected | 79 | 1.529 | 0.272 | 1.529 ± 0.272 |
| SFS Low | Unaffected | 28 | 1.6 | 0.217 | 1.600 ± 0.217 |
|  | Affected | 28 | 1.57 | 0.258 | 1.570 ± 0.258 |
| SFS High | Unaffected | 51 | 1.564 | 0.252 | 1.564 ± 0.252 |
|  | Affected | 51 | 1.506 | 0.28 | 1.506 ± 0.280 |
| Temporal Lobe | Unaffected | 47 | 1.59 | 0.219 | 1.590 ± 0.219 |
|  | Affected | 47 | 1.579 | 0.244 | 1.579 ± 0.244 |
| Frontal Lobe | Unaffected | 14 | 1.49 | 0.199 | 1.490 ± 0.199 |
|  | Affected | 14 | 1.375 | 0.275 | 1.375 ± 0.275 |
| Other Lobe | Unaffected | 18 | 1.625 | 0.317 | 1.625 ± 0.317 |
|  | Affected | 18 | 1.527 | 0.312 | 1.527 ± 0.312 |
| Nonlesional | Unaffected | 29 | 1.591 | 0.183 | 1.591 ± 0.183 |
|  | Affected | 29 | 1.535 | 0.208 | 1.535 ± 0.208 |
| Lesional | Unaffected | 50 | 1.57 | 0.265 | 1.570 ± 0.265 |
|  | Affected | 50 | 1.526 | 0.302 | 1.526 ± 0.302 |

**Reassessment of the DTI-ALPS index in Focal Epilepsy**

**ELECTRONIC SUPPLEMENTARY MATERIAL**

**Suppl. Table 1** Summary Statistics of DTI-ALPS Scores in Affected and Unaffected Hemispheres. Count (n) mean and standard deviation (SD) of DTI-ALPS scores for the unaffected and affected hemispheres across multiple clinical stratifications: overall epilepsy cohort, seizure frequency score (SFS) groups (“Control, SFS Low, SFS High”), lobar subgroups (“Temporal, Frontal, Other”), and etiological categories (Non-Lesional, Lesional). SFS Seizure Frequency Score, SD Standard deviation.

**Suppl. Fig. 1** Association of DTI-ALPS Score with White- and Gray Matter Volume. Relationship between ALPS values and total white- and gray matter volume normalized to total intracranial volume was assessed. (**a**) No significant associations were observed between DTI-ALPS and normalized TWM in the high and low SFS groups (control: *p* = 0.601, R² = 0.11; SFS-low: *p* = 0.653, R² = 0.06; SFS-high: *p* = 0.727, R² = 0.01).

(**b**) There were no significant associations between DTI-ALPS and normalized TGM in any group (control: *p* = 0.547, R² = 0.11; SFS-low: *p* = 0.724, R² = 0.05; SFS-high: *p* = 0.395, R² = 0.04). Scatterplots display linear regression fits for each group: control (blue), SFS-low (green), and SFS-high (red). Lines represent the predicted values from ordinary least squares regression, adjusted for age and sex. DTI-ALPS Diffusion tensor imaging analysis along the perivascular space, TWM Total white matter, TGM Total gray matter, TIV Total intracranial volume, SFS Seizure Frequency Score, n Sample size, β Coefficient.

**Suppl. Fig. 2** Association of DTI-ALPS Score with ILAE Outcome. No significant association between the DTI-ALPS score and ILAE outcome score dichotomized into 1 (i.e. no seizures after intervention) and ≥2 was found. Box plots display median (central line), interquartile range (box), and whiskers extending to 1.5 times the interquartile range. DTI-ALPS Diffusion tensor imaging analysis along the perivascular space, ILAE International league against epilepsy.
